# The Optimized Calculation Method for Insulin Dosage in an Insulin Tolerance Test (ITT): A Randomized Parallel Control Study

**DOI:** 10.3389/fendo.2020.00202

**Published:** 2020-04-09

**Authors:** Yuwen Zhang, Shouyue Sun, Huiying Jia, Yan Qi, Jie Zhang, Lin Lin, Yuhong Chen, Weiqing Wang, Guang Ning

**Affiliations:** ^1^Department of Endocrine and Metabolic Diseases, Ruijin Hospital North, Shanghai Jiao Tong University School of Medicine, Shanghai, China; ^2^State Key Laboratory of Medical Genomics, National Clinical Research Center for Metabolic Diseases, Collaborative Innovation Center of Systems Biomedicine, Ruijin Hospital, Shanghai Jiao Tong University School of Medicine, Shanghai, China; ^3^Shanghai Institute of Endocrine and Metabolic Diseases, Ruijin Hospital, Shanghai Jiao Tong University School of Medicine, Shanghai, China; ^4^Department of Endocrine and Metabolic Diseases, Ruijin Hospital, Shanghai Jiao Tong University School of Medicine, Shanghai, China

**Keywords:** insulin tolerance test, oral glucose tolerance test, insulin release test, insulin area under the curve, optimization coefficient

## Abstract

**Objective:** To explore the most suitable calculation method for insulin dosage in an insulin tolerance test (ITT) and to evaluate the clinical application value of the optimization coefficient (γ).

**Methods:** In this study, 140 adult patients with congenital growth hormone deficiency (GHD) or acquired hypopituitarism were randomized into the following two groups: the conventional group (*n* = 70) and the optimized group (*n* = 70). Oral glucose tolerance tests (OGTTs), insulin release tests (IRTs), and ITTs were conducted. For ITTs, insulin doses were the product of body weight (kg) and related coefficient (0.15 IU/kg for the control group and γ IU/kg for the optimized group, respectively). Notably, γ was defined as −0.034 + 0.000176 × AUC_INS_ + 0.009846 × BMI, which was based on our previous study.

**Results:** In the ITTs, the rate of achieving adequate hypoglycemia with a single insulin dose was significantly higher for the optimized group compared with the conventional group (92.9 vs. 60.0%, *P* < 0.001). The optimized group required higher initial doses of insulin (0.23 IU/kg). Meanwhile, the two groups did not differ significantly in their nadir blood glucose (1.9 vs. 1.9 mmol/L, *P* = 0.828).

**Conclusion:** This study confirmed that the proposed optimized calculation method for insulin dosage in ITTs led to more efficient hypoglycemia achievement, without increasing the incidence of serious adverse events.

## Introduction

The insulin tolerance test (ITT), which was developed in the late 1960s (1, 2), is widely accepted as the gold standard for the assessment of patients with growth hormone deficiency (GHD) (3). The standard intravenous insulin dosage administered in an ITT was 0.1–0.15 IU/kg body weight (4, 5). Adequate hypoglycemia is defined as a blood glucose nadir below 2.2 mmol/L or a blood glucose value below 2.6 mmol/L with a 50% reduction from baseline (6, 7). Previous studies have shown that blood glucose in some overweight or obese patients did not adequately decrease during ITTs, due to insulin resistance, thus repeated doses of insulin need to be administered (5, 8–11).

In our previous research, we analyzed 56 patients with congenital or acquired hypopituitarism. Oral glucose tolerance tests (OGTTs) and insulin release tests (IRTs) were conducted to evaluate glucose metabolic statuses of 56 patients with hypopituitarism. Homeostasis model of assessment for insulin resistance index (HOMA-IR), insulin sensitivity index (ISI), Area under curve of insulin (AUC_INS_), and area under curve of blood glucose (AUC_BG_) were calculated (Supplementary Table 1). All these patients had undergone the ITTs for evaluation of pituitary function. We found that, in ITTs, the initial dose of insulin did not adequately induce hypoglycemia (blood glucose value below 2.2 mmol/L or below 2.6 mmol/L with a 50% reduction from baseline) in 57.8% (32/56) patients (12). The ultimate insulin doses that induced hypoglycemia in ITTs were significantly inconsistent with the guideline-recommended ones (0.1–0.15 IU/kg body weight). Then, the main metabolic factors influencing the insulin dosage were analyzed (Supplementary Table 2). Multiple stepwise linear regression analysis found that AUC_INS_ and body mass index (BMI) were independent factors that affect the ultimate insulin dosage (Supplementary Table 3). Thus, we derived an equation to calculate the optimization coefficient γ (γ = −0.034 + 0.000176 × AUC_INS_ + 0.009846 × BMI) that could obtain the optimized insulin dosage (12).

In this study, we aimed to evaluate the clinical application value and safety of the optimized calculation method for insulin dosage in an ITT by comparing with the conventional one and to improve current ITT methods in clinical practice.

## Materials and Methods

### Subjects

This was a randomized parallel control study (ChiCTR1900023871, http://www.chictr.org.cn). We recruited patients suspected of having GHD or hypopituitarism based on their medical records from Ruijin Hospital North, Shanghai Jiao Tong University School of Medicine, China from June 2019 to September 2019. Patients were included in the study if they were ≥18 and ≤ 50 years old and displayed the following medical histories: (1) a history of congenital GHD diagnosed during childhood (or congenital hypopituitarism) or having clinical signs and symptoms of congenital GHD, (2) a history of hypothalamic-pituitary diseases with the clinical signs and symptoms of GHD, or (3) a history of craniocerebral tumor therapy (surgery or radiotherapy), wherein patients with sellar or pituitary tumors were beyond 6 months post-surgery. Patients were excluded if they were diagnosed with (1) diabetes, (2) severe hypertension (systolic blood pressure over 160 mmHg and/or diastolic blood pressure over 100 mmHg), (3) malignant tumor, (4) intracranial hypertension, (5) severe hepatic insufficiency (alanine transaminase > 3 times the upper limit of the normal range and/or aspartate aminotransferase > 3 times the upper limit of the normal range), (6) severe heart disease, (7) seizure, or (8) mental illness, or (9) they were currently pregnant and lactating. A total of 140 patients were enrolled in this study. The patients were randomized into the following two groups using Excel random numbers: the conventional group (*n* = 70) and the optimized group (*n* = 70). The study protocol was approved by the Committee on Human Research at Ruijin Hospital North, Shanghai Jiao Tong University School of Medicine, China. All the patients provided informed written consent to participate in the study.

### Methods

Detailed medical history and general clinical data, including age, gender, height, body weight, and BMI (weight divided by body height squared) were collected for all patients. Body weight was also used to calculate dose of insulin in ITTs. Venous blood samples were collected at rest in the morning after subjects had fasted overnight for measurement of the levels of blood glucose (BG), insulin (INS), glycated hemoglobin (HbA1c), serum insulin-like growth factor-1 (IGF-1), free thyroxine (FT_4_), testosterone (T), and estradiol (E_2_). Urinary free cortisol (UFC) was measured in a 24 h urine collection obtained before the dynamic tests.

Details related to prevalent hormone deficiencies and all medications at the time of testing, including hormone replacement, were recorded for each patient. No patient had received growth hormone (GH) replacement prior to the ITT. As the test is also a diagnostic test for adrenocorticotropic hormone-dependent hypoadrenalism, patients on chronic corticosteroid replacement therapy (generally 10–30 mg hydrocortisone per day) received their last dosage at 4 P.M. the day before testing, resulting in a drug restriction period of at least 16 h. All ITTs were conducted at 8 A.M. OGTTs and IRTs were conducted the day before ITTs. For each ITT, resuscitation equipment, 10 and 50% dextrose solutions and hydrocortisone were available should they be required. After completion of the ITT, patients were given a meal, after which the intravenous cannula was removed. Each ITT was performed at our unit by several experienced endocrine nurses and experienced endocrinologists.

### OGTT and IRT

1. All participants were instructed to fast for at least 10 h before the collection of blood samples. Five-point (0, 30, 60, 120, and 180 min) OGTT and IRT with a 75-g glucose load were performed. The 0, 30, 60, 120, and 180 min blood glucose (mmol/L; 0, 30, 60, 120, and 180 min BG, respectively) and 0, 30, 60, 120, and 180 min serum insulin (μIU/L; 0, 30, 60, 120, and 180 min INS, respectively) were measured.
2. Calculation of HOMA-IR (13)
  HOMA-IR = 0 min INS × 0 min BG/22.5.
3. Calculation of ISI
  ISI = log_10_ (0 min BG × 0 min INS).
4. Calculation of AUC_INS_
  AUC_INS_ = 0.5 × (0 min INS + 180 min INS) + 60 min INS + 120 min INS.
5. Calculation of AUC_BG_
  AUC_BG_ = 0.5 × (0 min BG + 180 min BG) + 60 min BG + 120 min BG.

### ITT

#### The Conventional Group (The Usual Treatment Group)

After fasting for at least 10 h, patients were fasted in bed, quiet, and conscious, and vital signs were monitored by an ECG monitor. A subcutaneous puncture was performed at the cubital vein to establish venous access with an indwelling vein tee. An intravenous bolus injection of regular insulin (Novo Nordisk China Pharmaceutical Co., Ltd.) at 0.15 IU/kg was performed. Venous blood samples for BG, GH, and cortisol were collected at −30, 0, 30, 45, 60, 90, and 120 min and capillary blood glucose was monitored at the same time. When the hypoglycemic symptoms occurred, these were also tested. The patient's vital signs were recorded at each of the above time points. Adequate hypoglycemia was defined as a blood glucose nadir below 2.2 mmol/L or a blood glucose value below 2.6 mmol/L with a 50% reduction from baseline. If this had not occurred by 45 min, a second 0.3 IU/kg insulin dose was administered and the record time was reset. Thereafter, no further doses were administered, and the procedure was abandoned.

#### The Optimized Group (The Experimental Group)

After fasting for at least 10 h, the patients were fasted in bed, quiet, and conscious, and their vital signs were monitored by an electrocardiograph (ECG) monitor. A subcutaneous puncture was performed at the cubital vein to establish venous access with an indwelling vein tee. An intravenous bolus injection of regular insulin (Novo Nordisk China Pharmaceutical Co., Ltd.) at γ IU/kg body weight was performed (γ = −0.034 + 0.000176 × AUC_INS_ + 0.009846 × BMI). Venous blood samples for BG, GH, and cortisol were collected at −30, 0, 30, 45, 60, 90, and 120 min, and capillary blood glucose was monitored at the same time. When the hypoglycemic symptoms occurred, these were also tested. The patient's vital signs were recorded at each of the above time points. Adequate hypoglycemia was defined as a blood glucose nadir below 2.2 mmol/L or a blood glucose value below 2.6 mmol/L with a 50% reduction from baseline. If this had not occurred by 45 min, a second 2 × γ IU/kg insulin dose was administered and the record time was reset. Thereafter, no further doses were administered, and the procedure was abandoned.

### Statistical Analysis

General characteristics were demonstrated according to the two groups. The Kolmogorov-Smirnov statistical test was used to assess data normality. Continuous variables were presented as the mean ± SD for normally distributed variables or medians (interquartile ranges) for the skewed variables. We log_10_-transformed the 0, 30, 60, 120, and 180 min INS, HOMA-IR, AUC_INS_, and AUC_BG_/AUC_INS_ to achieve a normal distribution. All the categorical variables were presented as numbers and proportions. We used unpaired Student's *t*-test or Mann-Whitney *U*-test to compare continuous variables and the chi-square test to compare categorical variables between the two groups. All analyses were performed with SPSS software version 22.0 (SPSS Inc., Chicago, IL, USA). Significance tests were two-tailed, and a *P* < 0.05 was considered statistically significant.

To estimate the group size, a pilot study was conducted for measuring the rate of achieving adequate hypoglycemia with a single dose of insulin in 10 patients with hypopituitarism who received an optimized dose of insulin. Adequate hypoglycemia was successfully induced with a single dose of insulin in eight of them. For our power calculation, we assumed an equal standard deviation in the conventional group and the optimized group. We wanted the capability to show a difference of 30% in the rate of achieving adequate hypoglycemia with a single dose of insulin between the two groups. With α = 0.05, two-tailed and a power of 85%, we needed 50 patients per group. Considering a compliance rate of 80 %, we asked 140 patients to participate in this study. The power analysis was performed with NCSS-PASS (version 11.0; NCSS, Kaysville, UT, USA).

## Results

### General Clinical Characteristics of the Two Groups

There were no differences in age, gender, BMI, ratio of congenital, and acquired cases, levels of pre-test IGF-1 between the two groups (*P* > 0.05) (Table 1). As patients in both groups have very low levels of pre-test IGF-1, they might have GHD. Meanwhile, the etiologic types of pituitary disease that make the patients suspicious of having GHD or hypopituitarism and pituitary hormone deficiencies of them were also listed in Table 1. There were also no differences in the ratio of pituitary hormone deficiencies between the two groups (*P* > 0.05). Table 2 showed clinical and laboratory control of other pituitary hormone deficiencies apart from GHD in the two groups. There were also no differences in levels of UFC and serum FT_4_ between the two groups (*P* > 0.05). Most of our patients' levels of serum *T* or *E*_2_ were below the lower limit of laboratory measurable value (*T* < 0.1 ng/mL, *E*_2_ < 20 pg/mL).

**Table 1:** Clinical characteristics of the two groups.

	**Conventional group**	**Optimized group**	***P*-value**
*N*	70	70	/
Age (year)	26.0 ± 7.8	28.1 ± 9.3	0.137
Gender (males/females)	57/13	60/10	0.076
BMI (kg/m^2^)	23.2 ± 4.7	24.2 ± 3.9	0.173
Congenital/acquired	42/28	42/28	1.000
**Underlying etiology (*****n*****)**			
Idiopathic GH deficiency	43	42	/
Pituitary adenoma	4	8	/
Germinoma	4	5	/
Craniopharyngioma	12	8	/
Rathke's cleft cyst	1	0	/
Lymphocytic hypophysitis	1	0	/
Langerhans cell histiocytosis	1	0	/
Other intracranial neoplasm	3	1	/
Other	1	6	/
Surgery (%)	28.6	24.3	0.702
Radiotherapy (%)	8.6	7.1	1.000
Pre-test IGF-1 (ng/ml)	50 (28–66)	44 (13-79)	0.951
GH deficiency (%)	98.6	95.7	0.620
ACTH deficiency (%)	81.4	75.7	0.537
TSH deficiency (%)	84.3	85.7	1.000
LH/FSH deficiency (%)	94.3	95.7	1.000
ADH deficiency (%)	14.3	7.1	0.274

*The data are the mean ± SD or median (quartile 1–3) for continuous variables and n (%) for categorical variables. SD, standard deviation; BMI, body mass index; GH, growth hormone; IGF-1, insulin-like growth factor-1; ACTH, adrenocorticotropic hormone; TSH, thyroid stimulating hormone; LH, luteinizing hormone; FSH, follicle stimulating hormone; ADH, antidiuretic hormone*.

**Table 2:** Clinical and laboratory control of other pituitary hormone deficiencies of the two groups.

	**Conventional group**	**Optimized group**	***P*-value**
*N*	70	70	/
UFC (μg/24 h)^a^	92.69 (56.40–168.36)	118.49 (69.29–157.05)	0.601
FT_4_ (pmol/L)^b^	9.48 (8.06–12.41)	9.75 (7.45–11.75)	0.554
*T* (male; ng/mL)^c^	<0.1 (<0.1–0.19)	<0.1 (<0.1–0.31)	NA
*E*_2_ (female; pg/mL)^d^	<20 (<20–24.0)	<20 (<20–22.3)	NA

*The data are median (quartile 1–3) for continuous variables. UFC, urinary free cortisol; FT_4_, free thyroxine; T, testosterone; E_2_, estradiol; NA, not applicable*.

a *The normal range of was 58–403 μg/24 h*.

b *The normal range of was 9.01–19.04 pmol/L*.

c *The normal range of was 1.75–7.81 ng/mL*.

d *The normal range of was 27–122 pg/mL for the follicular phase of the menstrual cycle*.

### Glycometabolism Characteristics of the Two Groups

There were no significant differences in terms of their 0, 30, 60, 120, and 180 min BG, 0, 30, 60, 120, and 180 min INS, HbA1c, HOMA-IR, ISI, AUC_BG_, AUC_INS_, and AUC_BG_/AUC_INS_ between the two groups. (*P* > 0.05; Table 3).

**Table 3:** Glycometabolic parameters of the two groups.

	**Conventional group**	**Optimized group**	***P*-value**
*N*	70	70	/
**OGTT**			
0 min BG (mmol/L)	4.8 ± 0.5	4.8 ± 0.6	0.911
30 min BG (mmol/L)	7.5 ± 1.4	7.3 ± 1.5	0.413
60 min BG (mmol/L)	6.7 ± 2.0	6.8 ± 2.2	0.786
120 min BG (mmol/L)	5.8 ± 1.6	6.0 ± 1.8	0.678
180 min BG (mmol/L)	5.2 ± 1.3	5.1 ± 1.2	0.604
**IRT**			
0 min INS (μIU/mL)	10.71 (5.85–15.20)	8.64 (6.29–13.60)	0.287
30 min INS (μIU/mL)	81.36 (56.48–123.25)	74.25 (46.09–132.55)	0.858
60 min INS (μIU/mL)	68.58 (46.35–124.50)	63.94 (37.19–118.93)	0.417
120 min INS (μIU/mL)	53.79 (32.79–89.38)	53.23 (27.99–108.93)	0.348
180 min INS (μIU/mL)	26.86 (14.70–52.23)	28.17 (15.27–49.37)	0.626
HbA1c (%)	5.5 ± 0.4	5.4 ± 0.4	0.934
HOMA-IR	2.15 (1.14–3.46)	1.82 (1.33–3.33)	0.272
ISI	1.66 ± 0.36	1.68 ± 0.41	0.682
AUC_INS_	137.21 (101.54–249.11)	130.91 (80.28–282.04)	0.454
AUC_BG_	17.58 ± 3.76	17.75 ± 4.38	0.805
AUC_INS_/AUC_BG_	0.12 (0.08–0.16)	0.12 (0.08–0.20)	0.963

*The data are the mean ± SD or median (quartile 1–3) for continuous variables and n (%) for categorical variables. SD, standard deviation, BG, blood glucose; INS, insulin; HbA1c, glycosylated hemoglobin. HOMA-IR, homeostasis model of assessment for insulin resistance index; ISI, insulin sensitivity index; AUC_INS_, area under curve of insulin; AUC_BG_, area under curve of blood glucose*.

### ITTs of the Two Groups

The actual insulin doses used in the two groups were shown in Table 4. The optimized group required higher initial doses of insulin compared to the conventional group (0.15 vs. 0.23 IU/kg, *P* < 0.001). The Ultimate insulin doses were also higher in the optimized group (0.21 vs. 0.24 IU/kg, *P* = 0.018). Among the ITTs, adequate hypoglycemia was successfully induced with a single dose of insulin in 107 patients, including 42 in the conventional group and 65 in the optimized group (Table 4). Among the remaining 33 patients, 15 patients (14 in the conventional group and 1 in the optimized group) achieved adequate hypoglycemia while 18 patients (14 in the conventional group and 4 in the optimized group) still failed after being given a repeated dose of insulin (Table 4). The rate of achieving adequate hypoglycemia with a single dose of insulin was significantly higher in the optimized group (65/70) than that in the conventional group (42/70) (92.9 vs. 60.0%, *P* < 0.001; Table 4). A similar finding was shown in patients who achieved adequate hypoglycemia with a single or repeated dose of insulin (94.3 vs. 80%, *P* = 0.021; Table 4). Of those who achieved adequate hypoglycemia with single dose of insulin, the two groups did not significantly differ with regard to their blood glucose nadirs (*P* = 0.828; Table 4). Moreover, among those with a repeated dose, there was also no difference in the nadir blood glucose between the two groups (*P* = 0.686; Table 4). In addition, the optimized group needed less time to achieve adequate hypoglycemia compare to the conventional group (*P* = 0.002). Duration of hypoglycemia after adequate hypoglycemia was achieved was not different between the two groups. No serious adverse reactions were recorded during the ITTs in either group (*P* > 0.05).

**Table 4:** ITT results of the two groups.

	**Conventional group**	**Optimized group**	***P-*value**
*N*	70	70	/
**Insulin dose (IU/kg)**			
Initial insulin dose	0.15 ± 0.01	0.23 ± 0.06	<0.001
Ultimate insulin dose	0.21 ± 0.10	0.24 ± 0.07	0.018
**Induction of adequate hypoglycemia (*****n*****, %)**			
Total	56 (80.0%)	66 (94.3%)	0.021
Single insulin dose	42 (60.0%)	65 (92.9%)	<0.001
Repeated insulin dose	14 (20.0%)	1 (1.4%)	/
Failed	14 (20.0%)	4 (5.7%)	0.021
**Blood glucose nadir (mmol/L)**			
Total	1.9 ± 0.4	1.8 ± 0.4	0.178
Single insulin dose	1.9 ± 0.4	1.9 ± 0.4	0.828
Repeated insulin dose	2.0 ± 0.4	1.8^*^	0.686
**Time needed to achieve adequate hypoglycemia (with single insulin dose;** ***n*****, %)**			
30 min	40 (57.1%)	58 (82.9%)	0.002
45 min	2 (2.9%)	7 (10.0%)	0.165
**Duration of hypoglycemia (*****n*****, %)**			
<15 min	48 (68.6%)	53 (75.7%)	0.451
15–30 min	7 (10.0%)	10 (14.3%)	0.606
30–45min	1 (1.4%)	3 (4.3%)	0.620
Serious adverse event (n)	0	0	/

*The data are the mean ± SD for continuous variables and n (%) for categorical variables*.

* *Only one case achieved adequate hypoglycemia with repeated insulin dose in the optimized group*.

## Discussion

In the present study, we demonstrated that the proposed personalized calculation method for an optimized insulin dosage in ITT led to a high rate of successful induction of adequate hypoglycemia with a single dose of insulin, without elevating the incidence of serious adverse events.

Based on the guidelines, regular insulin is administered at a dose of 0.1–0.15 IU/kg body weight in an ITT (4, 5). Recent studies and our previous clinical experience have shown that some patients did not develop adequate hypoglycemia with a single dose of insulin in ITTs, probably due to obesity or insulin resistance (5, 8–11). The possible underlying reason is that GH affects human metabolism, and glucolipid metabolism is the most prone to dysregulation because of GHD. It was found that patients with GHD present with increased TG and LDL-c and decreased HDL-c (14). In patients with GHD, increased adipose tissue and decreased muscle tissue lead to obesity and reduced exercise capacity, which lowers insulin sensitivity and subsequently causes insulin resistance and impaired glucose tolerance. Although our patients were with normal BG and HOMA-IR, their insulin sensitivity after a glucose load would change. A previous Asian study had derived a new index (ISI_OGTT_) calculated from body weight, fasting plasma glucose, AUC_BG_, AUC_INS_, and urinary glucose, which was considered to be more suitable than others in assessing insulin sensitivity in subjects with normal glucose tolerance (15). Perhaps this is why our patients were of relatively normal BG and HOMA-IR with underlying insulin resistance. Thus, repeated doses of insulin need to be administered, which will prolong the test time and influence the psychological state of the patients. It should be noted that patients with hypopituitarism usually loss some hyperglycemic hormones. The accumulation of insulin in the body may induce severe hypoglycemia, which may lead to pituitary apoplexy and even be fatal. Thus, we believe the insulin dosage used in an ITT should be personalized and increased in patients who are overweight, obese or with insulin resistance. Although several previous studies have suggested that the initial insulin dose in an ITT could be determined based on BMI, pre-test BG, with or without diabetes, acromegaly or suspected Cushing's syndrome, there have been no personalized calculation methods for insulin dosage until now (4, 5, 11, 16, 17).

To induce adequate hypoglycemia with a single dose of insulin and avoid repeated doses in ITT, we evaluated our personalized calculation method of insulin dosage in this study. In total, 140 patients were randomized into two groups. A comparison of general clinical characteristics was conducted and showed no significant difference between the two groups. Otherwise, the duration of GHD may also impact glycolipid metabolism, as well as insulin resistance, and therefore, differences in the ratios of congenital to acquired cases in the two groups were also analyzed but showed no significant differences between the two groups. The optimization coefficient (γ) was used to calculate the optimized insulin dosage in ITT, which resulted in a significantly higher rate of successful induction of adequate hypoglycemia with a single insulin dose compared to the conventional coefficient (92.9 vs. 60.6%, *P* < 0.001). The ultimate insulin doses used in ITTs of the optimized group were higher than the conventional group (0.21 vs. 0.24 IU/kg, *P* = 0.018). This is a bit odd that, unlike previous studies in western countries (4, 18), our patients seem to need much more insulin to achieve hypoglycemia in ITTs. The logical explanation for this is that, the relationship between the percent body fat and BMI is different among different ethnic groups and Asians have less muscle and more belly fat (19, 20). Body fat, especially visceral fat, is a major determinant of insulin resistance (21). Therefore, Asians should be at higher risk for insulin resistance than Caucasians with the same BMI.

As is known, ITT itself has the potential risk of triggering severe hypoglycemia and there have been reports of the death of pediatric subject (22). Patients with anterior pituitary hypofunction who lack several pituitary hormones usually have a very poor reserve of hyperglycemic hormones including GH and cortisol. They are also considered to be prone to hypoglycemia in ITTs. Therefore, it is important to achieve adequate hypoglycemia without adverse events by choosing an accurate insulin dose for an ITT. In terms of safety in our study, although the initial doses of insulin used in the optimized group were higher than the conventional group, the two groups did not differ with regard to their nadir blood glucose and the recovery time for hypoglycemia. Most of our patients in both groups had only adrenergic symptoms when achieved adequate hypoglycemia and all these patients recovered from hypoglycemia by drinking a glucose-rich beverage or just eating sweet foods. None of the patients had any serious adverse reactions to the test. We think that such results benefit from our personalized calculation method for insulin dosage. Most of our subjects were with congenital or acquired hypopituitarism at an early age. Long-term anterior pituitary hormone deficiency can lead to disorders of glucose and lipid metabolism, obesity with higher percentage of body fat and insulin resistance. Based on our previous research, our ITT methods takes into account both effectiveness and safety.

To the best of our knowledge, we are the first to investigate the personalized calculation method for insulin dosage in an ITT. However, there several limitations of this study that should be acknowledged. First, we did not include patients with diabetes mellitus in our study; thus, our findings would not be applicable to patients with diabetes. Second, since all patients in the present study were Chinese, it is unclear whether our results would be applicable to non-Chinese or non-Asian patients. Third, the optimized dosing requires the patient to undergo an OGTT before an ITT on another day, there is a little bit inconvenience to the patient having to undergo two tests on two spate days. However, for these Chinese patients, OGTTs and IRTs are required to assess their glucose metabolic status and insulin sensitivity. These additional tests are necessary to calculate the coefficient. Fourth, as we did not set another group that used less insulin, such as 0.1 IU/kg, so it was not known if a smaller dose of insulin would have been sufficient. But smaller doses of insulin should have a lower chance of inducing hypoglycemia according to our clinical experience. Furthermore, although we have performed power calculation (sample size calculation) in advance, this proposed calculation method should be further evaluated with larger samples in patients with different metabolic states or national races and in the future and studied for its clinical feasibility and safety.

In conclusion, our study confirmed that the proposed calculation method for an optimized insulin dosage in ITT led to a high rate of successful induction of adequate hypoglycemia with a single insulin dose without elevating the incidence of serious adverse events. This outcome promoted a more efficient and safer ITT.

## Data Availability Statement

The datasets generated for this study are available on request to the corresponding author.

## Ethics Statement

The studies involving human participants were reviewed and approved by Ethics Committee of Ruijin Hospital North Campus Shanghai Jiaotong University School of Medicine. The patients/participants provided their written informed consent to participate in this study.

## Author Contributions

YZ, SS, and WW designed the study, YZ, HJ, YQ, JZ, and LL collected the data. YZ and SS provided statistical analysis. YZ wrote the manuscript. WW, GN, SS, YC, and LL refined interpretation and the final manuscript. All authors were involved in writing the paper and had final approval of the submitted and published versions.

### Conflict of Interest

The authors declare that the research was conducted in the absence of any commercial or financial relationships that could be construed as a potential conflict of interest.
