# Supplementary material for: The Optimized Calculation Method for Insulin Dosage in an Insulin Tolerance Test (ITT): A Randomized Parallel Control Study
Source: Front Endocrinol (Lausanne). 2020 Apr 9;11:202. doi: 10.3389/fendo.2020.00202 (PMC7160329; doi:10.3389/fendo.2020.00202)
Supplement: Supplementary file 1 [file Data_Sheet_1.docx]

# Supplementary Data

**Supplementary Table 1.** Comparison of clinical data between patients with congenital and acquired pituitary function deficiency in our previous study.

|  | Congenital group | Acquired group | *P-*value |
| --- | --- | --- | --- |
| N | 33 | 23 | */* |
| Age (yr) | 26.42 ± 5.58 | 23.91 ± 8.34 | 0.026 |
| Gender (male / female) | 27 / 6 | 14 / 9 | 0.076 |
| BMI (kg/m^2^) | 22.46 ± 3.47 | 24.13 ± 3.71 | 0.992 |
| ALT (IU/L) | 48.88 ± 51.95 | 30.78 ± 14.84 | 0.024 |
| AST (IU/L) | 45.42 ± 38.52 | 35.09 ± 21.42 | 0.123 |
| ALP (IU/L) | 159.88 ± 78.16 | 130.26 ± 72.55 | 0.823 |
| γ-GT (IU/L) | 47.27 ± 68.83 | 30.48 ± 25.36 | 0.111 |
| BUN (mmol/L) | 4.41 ± 0.79 | 3.80 ± 1.06 | 0.415 |
| SCr (μmol/L) | 68.88 ± 10.18 | 70.36 ± 11.09 | 0.974 |
| SUA (μmol/L) | 349.09 ± 90.12 | 396.82 ± 92.25 | 0.329 |
| TG (mmol/L) | 2.22 ± 1.21 | 2.34 ± 1.27 | 0.760 |
| TC (mmol/L) | 5.35 ± 1.25 | 5.47 ± 1.53 | 0.103 |
| HDL-c (mmol/L) | 1.11 ± 0.29 | 1.19 ± 0.40 | 0.104 |
| LDL-c (mmol/L) | 3.76 ± 0.94 | 3.76 ± 1.20 | 0.118 |
| OGTT |  |  |  |
| 0 min BG (mmol/L) | 4.85 ± 0.57 | 4.76 ± 0.60 | 0.912 |
| 30 min BG (mmol/L) | 7.71 ± 1.47 | 7.77 ± 2.58 | 0.346 |
| 60 min BG (mmol/L) | 7.63 ± 2.65 | 8.16 ± 3.05 | 0.588 |
| 120 min BG (mmol/L) | 6.32 ± 2.65 | 7.11 ± 2.90 | 0.235 |
| 180 min BG (mmol/L) | 5.41 ± 1.48 | 5.92 ± 1.51 | 0.844 |
| IRT |  |  |  |
| 0 min INS (µIU/ml) | 9.52 (7.42 - 13.65) | 13.16 (5.43 - 19.29) | 0.240 |
| 30 min INS (µIU/ml) | 72.04 (35.77 - 133.30) | 129.10 (45.49 - 235.50) | 0.044 |
| 60 min INS (µIU/ml) | 59.86 (36.59 - 170.75) | 93.18 (24.98 - 207.10) | 0.237 |
| 120 min INS (µIU/ml) | 47.29 (32.66 - 113.20) | 70.93 (26.42 - 128.20) | 0.726 |
| 180 min INS (µIU/ml) | 30.12 (16.67 - 51.32) | 39.39 (20.55 - 79.20) | 0.833 |
| HbA1c (%) | 5.5 (5.2 - 5.6) | 5.5 (5.1 - 5.6) | 0.875 |
| HOMA-IR | 2.1 (1.5 - 3.0) | 2.8 (1.1 – 5.1) | 0.366 |
| ISI | 2.51 ± 0.42 | 2.51 ± 0.78 | 0.055 |
| AUC_INS_ | 139.34 (93.16 - 277.95) | 202.08.52 (90.19 - 370.20) | 0.700 |
| AUC_BG_ | 19.00 ± 4.98 | 20.61 ± 6.46 | 0.420 |
| AUC_BG_/AUC_INS_ | 0.13 (0.06 - 0.18) | 0.12 (0.05 - 0.17) | 0.063 |
| Ultimate insulin dose in ITT (IU/kg) | 0.20 (0.15 - 0.30) | 0.20 (0.17 - 0.29) | 0.409 |

The data are the mean ± SD or median (quartile 1 - quartile 3) for continuous variables and n (%) for categorical variables. SD, standard deviation. BMI, body mass index; ALT, alanine transaminase; AST, aspartate aminotransferase; ALP, alkaline phosphatase; γ-GT, γ-glutamyl transferase; BUN, blood urea nitrogen; SCr, serum creatinine; SUA, serum uric acid; TG, triglyceride; TC, total cholesterol; HDL-c, high density lipoprotein-cholesterol; LDL-c, low density lipoprotein-cholesterol; OGTT, oral glucose tolerance test; BG, blood glucose; IRT, insulin release test; INS, insulin; HbA1c, glycated hemoglobin; HOMA-IR, homeostasis model of assessment for insulin resistance index; ISI, insulin sensitivity index; AUC_INS_, area under curve of insulin; AUC_BG_, area under curve of blood glucose.

**Supplementary Table 2.** Correlation analysis for ultimate insulin dose in ITT and various parameters in 56 patients.

| Parameter | Mean±SD | correlation coefficient (*r*) | *P*-value |
| --- | --- | --- | --- |
| Age (yr) | 25.39±6.89 | ﹣0.125 | 0.359 |
| Gender (male/female) | 41/15 | 0.022 | 0.874 |
| BMI (kg/m^2^) | 23.14 ± 3.63 | 0.532 | < 0.001 |
| ALT (IU/L) | 41.44 ± 43.56 | 0.249 | 0.064 |
| AST (IU/L) | 41.17 ± 32.75 | 0.113 | 0.405 |
| ALP (IU/L) | 147.71 ± 76.65 | 0.095 | 0.485 |
| γ-GT (IU/L) | 40.37 ± 55.52 | 0.170 | 0.209 |
| BUN (mmol/L) | 4.16 ± 0.94 | ﹣0.368 | 0.006 |
| SCr (μmol/L) | 69.47 ± 10.47 | 0.019 | 0.889 |
| SUA (μmol/L) | 368.18 ± 93.16 | 0.463 | < 0.001 |
| TG (mmol/L) | 2.27 ± 1.22 | 0.225 | 0.095 |
| TC (mmol/L) | 5.39 ± 1.35 | 0.130 | 0.339 |
| HDL-c (mmol/L) | 19.77 ± 139.49 | 0.255 | 0.058 |
| LDL-c (mmol/L) | 3.76 ± 1.04 | 0.160 | 0.238 |
| 0 min BG (mmol/L) | 4.81 ± 0.58 | 0.366 | 0.006 |
| 30 min BG (mmol/L) | 7.73 ± 1.92 | 0.074 | 0.59 |
| 60 min BG (mmol/L) | 7.85 ± 2.80 | 0.407 | 0.002 |
| 120 min BG (mmol/L) | 6.64 ± 2.39 | 0.364 | 0.006 |
| 180 min BG (mmol/L) | 5.62 ± 1.49 | 0.156 | 0.255 |
| 0 min INS (µIU/ml) | 11.53 (6.39 - 16.73) | 0.553 | < 0.001 |
| 30 min INS (µIU/ml) | 78.57 (40.83 - 163.25) | 0.432 | 0.001 |
| 60 min INS (µIU/ml) | 75.98 (36.25 - 188.05) | 0.507 | < 0.001 |
| 120 min INS (µIU/ml) | 63.37 (29.10 - 118.23) | 0.604 | < 0.001 |
| 180 min INS (µIU/ml) | 31.51 (17.76 - 67.01) | 0.409 | 0.002 |
| HbA1c (％) | 5.5 (5.2 - 5.6) | 0.117 | 0.424 |
| HOMA-IR | 2.3 (1.3 - 3.4) | 0.563 | < 0.001 |
| ISI | 2.51 ± 0.59 | 0.416 | 0.001 |
| AUC_INS_ | 171.52 (92.26 - 326.86) | 0.610 | < 0.001 |
| AUC_BG_ | 19.66 ± 5.63 | 0.406 | 0.002 |
| AUC_BG_/AUC_INS_ | 0.13 (0.06 - 0.18) | ﹣0.394 | 0.003 |

The data are the mean ± SD or median (quartile 1 - quartile 3) for continuous variables.

**Supplementary Table 3.** Multiple stepwise linear regression analysis based on ultimate insulin dose in ITT (IU/kg) as dependent variable.

| Model |  | Regression coefficient | Standard regression coefficient | Standard error | *t* | *P* |
| --- | --- | --- | --- | --- | --- | --- |
| 1 | Constant | 0.175 |  | 0.015 | 11.636 | < 0.001 |
|  | AUC_INS_ | 0.000246 | 0.626 | 0.000042 | 5.802 | 0.001 |
| 2 | Constant | -0.034 |  | 0.067 | - 0.508 | 0.612 |
|  | AUC_INS_ | 0.000176 | 0.448 | 0.00045 | 3.915 | < 0.001 |
|  | BMI | 0.009846 | 0.363 | 0.003109 | 3.167 | 0.003 |
